# Correction: APTES assisted surface heparinization of polylactide porous membranes for improved hemocompatibility

**DOI:** 10.1039/c8ra90029a

**Published:** 2018-04-17

**Authors:** Jinglong Li, Fu Liu, Xuemin Yu, Ziyang Wu, Yunze Wang, Zhu Xiong, Jidong He

**Affiliations:** Ningbo Institute of Materials Technology & Engineering, Chinese Academy of Sciences Ningbo 315201 P. R. China fu.liu@nimte.ac.cn +86-574-86685256; Qingdao University of Science & Technology Qingdao 266000 P. R. China

## Abstract

Correction for ‘APTES assisted surface heparinization of polylactide porous membranes for improved hemocompatibility’ by Jinglong Li *et al.*, *RSC Adv.*, 2016, **6**, 42684–42692.

The authors regret that affiliation b was incorrect in the original version of the manuscript. The correct affiliation is presented herein.

The Royal Society of Chemistry apologises for these errors and any consequent inconvenience to authors and readers.

## Supplementary Material

